# Critical Review on the Public Health Impact of Norovirus Contamination in Shellfish and the Environment: A UK Perspective

**DOI:** 10.1007/s12560-017-9279-3

**Published:** 2017-02-07

**Authors:** Francis Hassard, Jasmine H. Sharp, Helen Taft, Lewis LeVay, John P. Harris, James E. McDonald, Karen Tuson, James Wilson, David L. Jones, Shelagh K. Malham

**Affiliations:** 1 Centre for Applied Marine Sciences, School of Ocean Sciences, Bangor University, Menai Bridge, Anglesey LL59 5AB UK; 2 Natural Resources Wales, Ty Cambria, Cardiff, CF24 0TP UK; 3 School of Environment, Natural Resources & Geography, Bangor University, Bangor, Gwynedd LL57 2UW UK; 4 NIHR Health Protection Research Unit in Gastrointestinal Infections, University of Liverpool, Liverpool, L69 3GL UK; 5 School of Biological Sciences, Bangor University, Bangor, Gwynedd LL57 2UW UK; 6 Bangor Mussel Producers Ltd., Victoria House, Plas Llwyd Terrace, Bangor, Gwynedd LL57 1UB UK

**Keywords:** Aquaculture, Food safety, Norovirus, Norwalk, Oyster, Shellfish

## Abstract

We review the risk of norovirus (NoV) infection to the human population from consumption of contaminated shellfish. From a UK perspective, risk is apportioned for different vectors of NoV infection within the population. NoV spreads mainly by person-to-person contact or via unsanitary food handling. NoV also enters the coastal zone via wastewater discharges resulting in contamination of shellfish waters. Typically, NoV persists in the marine environment for several days, with its presence strongly linked to human population density, wastewater discharge rate, and efficacy of wastewater treatment. Shellfish bioaccumulate NoV and current post-harvest depuration is inefficient in its removal. While NoV can be inactivated by cooking (e.g. mussels), consumption of contaminated raw shellfish (e.g. oysters) represents a risk to human health. Consumption of contaminated food accounts for 3–11% of NoV cases in the UK (~74,000 cases/year), of which 16% are attributable to oyster consumption (11,800 cases/year). However, environmental and human factors influencing NoV infectivity remain poorly understood. Lack of standard methods for accurate quantification of infective and non-infective (damaged) NoV particles represent a major barrier, hampering identification of an appropriate lower NoV contamination limit for shellfish. Future management strategies may include shellfish quality assessment (at point of harvest or at point of supply) or harvesting controls. However, poor understanding of NoV inactivation in shellfish and the environment currently limits accurate apportionment and risk assessment for NoV and hence the identification of appropriate shellfish or environmental quality standards.

## Introduction

Human NoV is a highly infectious gastrointestinal infection with an incubation period of 10–50 h (ACMSF 2014). NoV illness is characterised by nausea, vomiting and/or watery non-bloody diarrhoea, abdominal or general muscle pain, headache, and chills or fever (Glass et al. 2009; HPA 2004) and can result in dehydration, particularly in locations with poor drinking water quality (Mattner et al. 2006). NoV infection is typically self-limiting (12–48 h) usually without the requirement for medical treatment for recovery (FSA 2015; HPA 2004). Vulnerable people such as the elderly, immunocompromised or the very young can require additional care (Harris et al. 2008). Outbreaks of NoV frequently occur in high-density settings such as care homes, hospitals, and cruise ships; however, outbreaks in restaurants, hotels, holiday camps and through consumption of contaminated drinking water are also commonplace (Heijne et al. 2009; Werber et al. 2009).

The majority of transmission events occur through person-to-person contact, via contact with contaminated surfaces or environments, while other vehicles of infection such as food or water have also been identified (Mathijs et al. 2012; Matthews et al. 2012). NoV typically spreads through contact with or ingestion of faeces or vomit from carriers or infected individuals (HPA 2004). The proportion of NoV derived from different routes is poorly defined due to high levels of uncertainty surrounding epidemiological estimates resulting from a high underreporting ratio of infection and difficulties identifying the vehicle of infection (ACMSF 2014; Tam et al. 2014).

Access to sustainable sources of protein has become one of the major challenges of modern society. In this context, the cultivation of bivalve shellfish offers one potential solution while simultaneously promoting environmental and economic sustainability in coastal regions (Dumbauld et al. 2009). In 2010, the global shellfish aquaculture industry had a value of €1.1 billion (STECF 2013) and was worth ~€15.2 million to the UK (Seafish Guide to Aquaculture 2013). The contamination of bivalve shellfish with enteric pathogens such as human norovirus (NoV) in the harvesting area, however, is recognised as a hazard to human health and the continued growth of sustainable aquaculture practices (Lees 2010; Lowther et al. 2010a, 2012a). Current risk assessment and food hygiene regulation rely on bacterial indicators of faecal pollution (e.g. *Escherichia coli* and intestinal enterococcus) in shellfish (EC 2004, 2008). However, bacterial indicators often do not adequately represent the risk from enteric human viral pathogens, such as norovirus (NoV) (Ang 1998; Baert et al. 2011). Despite recent advances in methodologies for the cultivation of human NoV (Robinson and Pfeiffer 2014; Jones et al. 2015), at present NoV currently cannot be reliably cultivated for the routine analysis of foodstuffs or environmental samples. Viral pathogen detection in shellfish is based on molecular diagnostics such as reverse transcription qPCR (RT-qPCR). A standard method for quantification of viral pathogens from foodstuffs (ISO/TS 15216-1:2013; ISO/TS 15216-2:2013) has improved comparative viral quantification between laboratories (Anonymous 2013a, b).

In this critical review, we examine contributions of different sources of contamination of NoV to the environment and examine proposed methods to monitor and regulate these sources. We examine the role that shellfish play as a primary vector of NoV, and apportion their contribution to secondary and tertiary infections, using the UK as an example. We examine the methods for assessing viral titre and discuss the application of viral standards on shellfish aquaculture, as well as a range of options for changes to regulatory controls and production area management that may help mitigate human health risk from viral pathogens in shellfish.

## NoV Abundance and Distribution in Estuarine and Coastal Waters

### Point Source NoV Discharges into the Environment

**Fig. 1:**
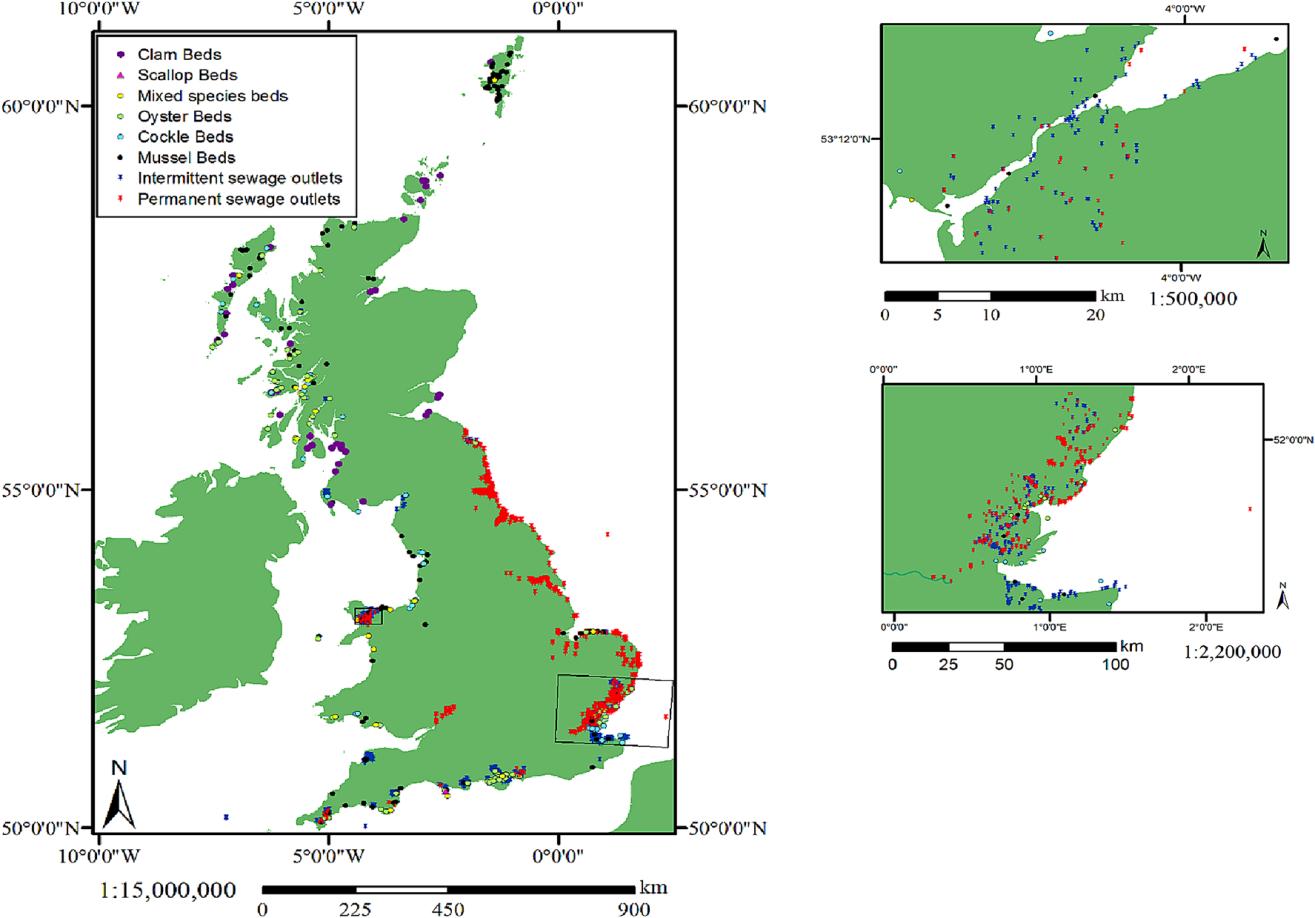
Shellfish areas and sewage outflow distribution in UK waters. *Insert top*—Northwest Wales coast; *inset bottom*—Southeast England data adapted from sanitary survey data (Kershaw et al. 2012)

Shellfisheries are at risk of NoV through contamination with human faecal matter which arises from effluents of wastewater treatment works (WWTWs), storm overflows (SOs), combined sewage overflows (CSOs), septic tanks or boats (Haramoto et al. 2005; Mathijs et al. 2012; EURL 2014b). Intermittent discharges offer a greater risk in areas with high average rainfall/runoff or subject to flashy storm events (Riou et al. 2007). Viral particles can persist in the marine environment and be transported (>10 km) representing a possible risk to shellfish production areas (Flannery et al. 2013; Winterbourn et al. 2016). WTWWs are currently not designed to be effective at removing viruses such as NoV. Typically, tertiary treatments (i.e. UV or membrane filtration) have been shown not to reduce NoV surrogates to a non-infectious level (Palfrey et al. 2011). Similarly, infrequent outbreaks linked to recreational bathing waters have been recorded (Maunula et al. 2004). In open coastal environments, NoV concentrations are expected to be lower compared to rivers and estuaries (Wyn-Jones et al. 2011). In the UK, the highest number of wastewater discharges tends to be located in areas of greatest population (Fig. 1). Shellfish beds are located in areas of both high continuous and intermittent wastewater discharges (Fig. 1).

The principal NoV contamination risk comes from WWTWs with a continuous discharge and serving >80,000 population equivalents (PE; DEFRA 2015). In 2014, there were 336 WWTWs in the UK and 5417 WWTWs in Europe with a consented flow >80,000 PE (designated at peak flow conditions). Of the UK works, <30 sites have continuous discharges of >80,000 populations equivalents in oyster harvesting zones mainly in the Southeast of England (DEFRA 2015; Fig. 1). However, the impact of the different sewage treatment processes employed across the WWTWs (CSOs and SOs) on viral loading remains uncertain (Pommepuy et al. 2009). The highest probability of contamination arises from untreated or primary-treated sewage—primary treatment typically results in ~2 log_10_ reduction in viral loading (Palfrey et al. 2011). The efficacy of secondary WWTW at reducing NoV loading varies considerably between treatment types (Campos and Lees 2014). Tertiary waste treatment can be applied to improve the microbiological quality of discharge; however, the efficacy at reducing viral loading to non-infectious levels requires further research (Flannery et al. 2013; Palfrey et al. 2011). Also, given the significant costs of such upgrades and current absence of regulatory controls on viruses in wastewater discharges, investment in tertiary treatment may be considered unjustifiable unless significant human health risk or benefit can be demonstrated. Membrane bioreactors (MBRs) have reported a 1.84–5 log_10_ reduction in viral titre (Armon et al. 2007; Doré et al. 2013). Modelling suggested that UV treatment results in a ~2 log_10_ reduction (Aquatic Water Services Ltd. 2014). The reliability of UV disinfection is dependent on the efficiency of upstream processes and applying a suitable wavelength and dose, for a sufficient period of time (Campos and Lees 2014). Although current technology cannot discern the infectivity of viruses (Lees 2010), storing wastewater for 4 weeks at ambient temperature may reduce infectious viruses implying that these treatments could reduce NoV viability (Tian et al. 2012), though this is unlikely to be practical. Small private septic tanks also represent a risk directly into shellfish waters, with approximately 4% of the UK population served by off-grid treatment facilities (DEFRA 2012b) that discharge onto land or tributaries and are considered diffuse pollution at the catchment scale (DEFRA 2015).

### Diffuse NoV Discharges into the Environment

Norovirus contamination from shipping and recreational boating represents a limited, seasonal NoV contamination risk (Kershaw et al. 2012; ACMSF 2014). Overboard discharges from small boats are currently unregulated in the UK (ACMSF 2014; Campos and Lees 2014), although regulations are in place preventing large vessels (>400 Gt, or <400 Gt which are certified to carry more than 15 persons) from direct discharge of untreated effluents (Kershaw et al. 2012). In urban areas, sewer misconnections are an additional concern where foul pipes from toilets can be connected to surface water outfalls or streams instead of the sewer. The most frequent domestic misconnections are ‘grey water’ types such as washing machines, sinks, and dishwashers accounting for ~80% of the misconnections in UK catchments (Ellis and Butler 2015).

The use of sludge solids (from the sewage treatment process), as applied to land may pose a potential risk of spreading NoV (DEFRA 2012). However, the application of untreated sewage sludge to all food and non-food crops has been restricted in the UK since 2005 (ADAS 2001) reducing to a minimum the viral contamination of shellfish waters from the application of sewage sludge to land. In addition, 75% of the sludge destined for land is treated by anaerobic digestion, potentially reducing viral titres by ~99% (DEFRA 2012). In contrast, other methods such as heat, lime treatment or solar drying treatments are thought to result in complete pathogen deactivation (ACMSF 2014; ADAS 2001).

### Sources of NoV Affecting Shellfisheries

Typically, shellfish harvesting and production areas are found within estuaries and coastal zones (HPA 2004), which have variable freshwater input from rivers and point source discharges, which impacts upon the distribution and persistence of NoV (Table 1). Previous research efforts have largely focused on detecting the presence/absence of NoV, while a limited number of studies have also quantified the concentration/loading, persistence over time or distribution of NoV in aquatic environments (Table 1). Based on the data available, detection of NoV in marine water samples was variable between sites and genogroups. In studies undertaken in Brazil and China, NoV genogroup I (GI) accounted for 0–67% (where detected) of the total NoV detected, while in contrast NoV genogroup II (GII) accounted for 8–100% (where detected) (Marques Souza et al. 2012; Victoria et al. 2010a; Yang et al. 2012). Wyn-Jones et al. (2011) detected NoV in 16.4% of marine water samples (GI + GII; 79/482 samples), with GI detected in 7.9% (38/482) of samples and GII detected in 8.5% (41/482) of samples in Europe (Table 1). The minimum reported viral concentration for GI was 2.6 ± 1.7 log_10_ gc l^−1^, and for GII, 2.7 ± 1.8 log_10_ gc l^−1^ (detected in Hong Kong), although these values are subject to variability due to spatial and seasonal outbreaks (Yang et al. 2012). Maximum reported concentrations were 5.5 log_10_ gc l^−1^ for GI and 6.1 log_10_ gc l^−1^ for GII in Brazil (Marques Souza et al. 2012; Moresco et al. 2012).

**Table 1:** Prevalence and concentration of norovirus in marine, estuarine, and riverine waters

Environment	Location	Prevalence % (samples)		Concentration (log_10_ gc l^−1^)		Reference
		GI	GII	GI	GII	
Marine	Brazil	0% (0/4)	25.0% (1/4)	–	6.1	Marques Souza et al. (2012)
	Brazil	7.5% (10/132)	4.5% (6/132)	4.3–5.5	–	Moresco et al. (2012)
	Brazil	8.3% (1/12) to 16.7% (2/12)	8.3% (1/12)	–	–	Victoria et al. (2010a, b)
	China	66.7% (4/6)	100% (6/6)	2.6 ± 1.7 to 3.6 ± 2.1	2.7 ± 1.8 to 3.6 ± 2.6	Yang et al. (2012)
	Europe	7.9% (38/482)	8.5% (41/482)	–	–	Wyn-Jones et al. (2011)
	Italy	30.0%^b^	–	–	–	Wyn-Jones et al. (2011)
	Italy	–	16.3%^b^	–	–	Wyn-Jones et al. (2011)
Estuarine	Brazil	8.3% (1/12)	0% (0/12)	–	–	Victoria et al. (2010a, b)
	France	7.0% (5/70)	24.0% (17/70)	3.4 (2.1–3.7)	4.3 (2.6–5.0)	Zakhour et al. (2010)
	Mexico	–	–	–	–	Hernandez-Morga et al. (2009)
	N. Zealand	60.0% (9/15)	100% (15/15)	1.7 (1.7–1.8)	2.9 (2.4–3.4)	Hewitt et al. (2013)
Riverine	Brazil	41.7% (5/12)	8.3% (1/12)	–	–	Victoria et al. (2010a, b)
	Europe	–	–	–	–	Wyn-Jones et al. (2011)
	Japan	47.0% (28/60)	30.0% (18/60)	–	–	Kitajima et al. (2010)
	Japan	–	–	–	–	Ueki et al. (2005)
	N. Zealand	37.1% (13/35) to 95.2% (20/21)	37.1% (13/35) to 95.2% (20/21)	1.7 (1.7–1.7)	2.0 (1.7–2.4)	Hewitt et al. (2013)
	S. Africa	12.5% (3/24) to 23.8% (5/21)	4.8% (1/21) to 23.5% (25/106)	2.9 (1.7–3.3)	2.6 (2.0–2.9)	Mans et al. (2013)
	S. Korea	20.0% (5/25)	56.0% (14/25)	–	–	Park et al. (2011)
	Netherlands	–	15.0%^b^	–	–	Wyn-Jones et al. (2011)
	UK	10.0%^b^	–	–	–	Wyn-Jones et al. (2011)

^a^Log_10_ genome copies or viral particles per litre of water

^b^Number of samples not stated

In estuarine sites, NoV GI is frequently detected (7–60% of sites sampled in New Zealand; Hewitt et al. 2013), while detection of GII appears more variable with detection levels ranging from 0 to 100% (Victoria et al. 2010a; Hewitt et al. 2013). However, in some estuarine environments, NoV GII abundance was greater than GI, with minimum values of 1.7 log_10_ gc l^−1^ for GI, and 2.4 log_10_ gc l^−1^ for GII (Hewitt et al. 2013). The greatest reported NoV abundance was 3.7 log_10_ and 5.0 log_10_ gc l^−1^ for GI and GII, respectively, in France (Zakhour et al. 2010). In river samples, GI had a higher abundance than GII (Table 1). Overall detection of NoV in European rivers was 6.3%, based on 928 samples (Wyn-Jones et al. 2011). The concentration of NoV was similar for the two genogroups (difference ~0.3 log_10_ gc l^−1^); however, an agricultural catchment had lower NoV (difference ~0.9 log_10_ en l^−1^) than an urban-dominated catchment suggesting different loadings or persistence between these environments.

### NoV Persistence in Water

Enteric viruses, including NoV, typically persist for several days in water; one study has suggested that 4–6 days be used for risk assessment purposes based on the time taken to achieve a 90% reduction in NoV genome copies (T_90_) (Aquatic Water Services Ltd. 2014). However, environmental persistence of up to 30 days has also been reported (Pommepuy et al. 2004). For comparison, faecal indicator bacteria (FIB) persist for typically between 0.3 and 6.6 days in the day and night, respectively, based on established decay rates (Whitehead et al. 2016).

Factors such as elevated temperatures and exposure to UV radiation tend to have a negative effect on both FIB survival and viral persistence in the water column (Aquatic Water Services Ltd. 2014). Effective in vitro NoV cultivation has only recently been demonstrated by Ettayebi et al. (2016). Therefore, studies on factors which govern environmental persistence and/or abrogation of infectivity have not been undertaken. Typically, monitoring programmes are based on detection of NoV genome copies only. The seasonality associated with NoV detection in water can be linked to infection rate within the population, lower solar irradiation and temperature (Lopman et al. 2009), and higher water turbidity (Lowther et al. 2012a). In the northern hemisphere, NoV is therefore most prevalent in estuarine and riverine waters between October and April (Haramoto et al. 2005; Kitajima et al. 2010; Mans et al. 2013; Moresco et al. 2012). NoV prevalence is also positively correlated with sites close to inputs of human faecal matter (Ueki et al. 2005; Victoria et al. 2010b; Wyn-Jones et al. 2011) with a subsequent reduction due to dilution with increasing distance from the source (Kitajima et al. 2010). However, NoV has been observed up to 10 km from wastewater discharge points, indicating high survival rates of NoV, persistence in the environment and widespread contamination of the coastal zone (Aquatic Water Services Ltd. 2014; Winterbourn et al. 2016).

## Epidemiology of NoV in the UK

NoV is the commonest cause of infectious intestinal disease (IID) in the UK, with an estimated three million cases each year based on sporadic (non-outbreak) infections (FSA 2000; Phillips et al. 2010) and secondary infections (Tam et al. 2012a, b, c; Tam et al. 2014). Given the nature of the illness and public health advice, persons infected with NoV seldom contact medical services, resulting in low reporting rates. It is estimated that only one case of NoV is reported to national surveillance for every 288 cases (largely outbreak-related cases) in the community (Tam et al. 2012c). Outbreak-related (non-sporadic) cases may account for a further 10,000 to 16,000 cases annually, with 15,529 laboratory-confirmed cases in 2010 and 9,382 in 2013, respectively (FSA 2014).

The estimated total number of cases in the UK is 2.65 million per annum (outbreak cases in 2013 × underreporting ratio). However, this number should be treated with caution due to coinfections with other pathogens and the transmission by asymptomatic carriers (Phillips et al. 2009; 2010), or inefficient data submitted to the UK national surveillance could result in an over-estimate of the influence of NoV on IID (Tam et al. 2012b, c). Typically, public perception is that transmission of IID’s is due to consumption of contaminated seafood. This is discussed in relation to the epidemiology of the disease.

### Person-to-Person Transmission

Within Europe, typically, 74 to 85% of reported NoV outbreaks are caused by direct spread between humans (Mathijs et al. 2012), while a study in the Netherlands estimated 55% (42% to 88%) of all NoV cases occurred by this route (Havelaar et al. 2008). In comparison, 85% of outbreaks in England and Wales originated from person-to-person transmission (Lopman et al. 2003). Person-to-person transmission is common in enclosed settings where isolation of infected individuals is challenging. Food can be an important vector for initial disease introduction (HPA 2004; HPA 2007). In the UK, 79% of reported outbreaks took place in health care institutions, while 43 deaths, during 38 outbreaks, occurred in hospitals and residential care facilities (Lopman et al. 2003).

### Water-borne Transmission

Water-borne transmission is less common than NoV infections associated with food consumption, and most commonly occurs via ingestion of contaminated drinking water or recreational/bathing water (Baert et al. 2009; Werber et al. 2009). Overall, water-borne transmission of NoV represents a minor route of infection in the UK. Treatment of water intended for consumption in the UK is considered to be effective at eliminating the risk of NoV infection (Gormley et al. 2011).

### Food-Associated NoV Infection

Estimates of food-associated transmission burden vary widely, from 11% to 25% at the global scale (Table 2), and from 2.7% to 11% in the UK (estimated at ~ 74,000 cases per year) (Adak et al. 2002; Tam et al. 2014). Comparing food-borne transmission data between countries is challenging due to different food consumption behaviour, transmission pathways, data collection, and reporting methods (ACMSF 2014). Annual community cases of 69,628 in 1995, and 61,584 each year between 1996 and 2000 have been reported previously (Adak et al. 2002, 2005). The proportion of food-borne cases, therefore, is low compared with non-food-borne infections. Despite the number of NoV cases, < 1% of food-associated NoV infected individuals are hospitalised (Tam et al. 2014) which represents a burden of ~ 400 cases per year (Table 3).

**Table 2:** Estimated food-associated norovirus transmission rates, by country

Country	Proportion of infections that are food-associated (%)	Reference
Australia	25	Hall et al. (2005)
France	14	Vaillant et al. (2005)
The Netherlands	17	Havelaar et al. (2008)
UK	11	Adak et al. (2002)
USA	25	Scallan et al. (2011)

Adapted from: ACMSF (2014)

**Table 3:** Estimated community cases, GP consultations, and hospital admissions related to food-associated norovirus transmission in the UK using different approaches (2009)

Modelling approach	Community cases	GP consultations	Hospital admissions
	Cases (95% CI)	Cases (95% CI)	Cases (95% CI)
Monte Carlo	73,420	3240	470
	(50,320–104,000)	(1985–5162)	(270–779)
Bayesian	74,100	3276	332
	(61,150–89,660)	(2240–4729)	(248–440)

Adapted from: Tam et al. (2014)

## Scale of Human Health Risk in Respect to NoV from Shellfish

The bioaccumulation and persistence of NoV in shellfish is influenced by viral concentration in the surrounding environment, shellfish metabolic activity and the effects of any post-harvest treatments applied to the shellfish (Campos and Lees 2014). Oyster tissues can bioaccumulate coliphage to levels ~ 100 times higher than in the surrounding waters (Burkhardt and Calci 2000; Drouaz et al. 2015) suggesting that under optimal conditions, NoV can rapidly bioaccumulate (Baker et al. 2010). Further, NoV can remain in shellfish tissues for ≤ four weeks after a pollution event (Campos and Lees 2014), even after the pathogen is no longer present in the surrounding environment (≤ 12 days, Asahina et al. 2009). This finding has implications for the success of post-harvest purification treatments which have been optimised for bacterial depuration.

The majority harvested shellfish in the UK are sold for human consumption after cooking which can reduce NoV surrogates in shellfish to non-infectious levels (e.g. 90 °C for 3 min, Flannery et al. 2014). NoV infections, where shellfish are the implicated vector, are commonly associated with the consumption of raw or undercooked bivalve shellfish. Raw shellfish are implicated in over half (58.4%) of viral disease outbreaks from shellfish consumption worldwide (Alfano-Sobsey et al. 2012; Bellou et al. 2013). In the UK, native oysters (*Ostrea edulis*) and Pacific oysters (*Crassostrea gigas*) are sold for consumption raw, and as such pose an increased NoV risk to consumers compared to cooked shellfish (Lees 2010). An investigation into a peak in northern European NoV outbreaks in 2010 associated with oyster consumption implicated UK oysters (Westrell et al. 2010), suggesting that oyster consumption is important for NoV risk characterisation.

### NoV Abundance, Distribution, and Persistence in Shellfish

In a UK study of 39 oyster production areas, NoV was detected in 76.2% of shellfish samples tested. Of these, GI was detected at 20.9% of sites, GII detected at 7.7% of sites and GI and GII both detected at 47.6% of the sites (Lowther et al. 2012a; FSA 2011; CEFAS 2011a, b; CEFAS 2014). The concentration of NoV varied widely, with maxima of 16,507 gc g^−1^ for GI and 18,024 gc g^−1^ for GII. High concentrations were relatively infrequent, with most samples containing detectable NoV levels below the limit of quantification (LOQ) of 100 gc g^−1^ (Lowther et al. 2012a). NoV shows a marked seasonal pattern, with maximum contamination levels observed in the winter months, and minimum levels in summer (FSA 2011; Keaveney et al. 2009; Woods and Burkhardt III 2010). CEFAS (2011b) suggests that NoV is negatively linked to temperature as contamination from oysters is derived from contaminated human faecal matter, which principally occurs due to NoV outbreaks in winter months (Lopman et al. 2009). Detection rates for oysters were 90% in winter (October to March), compared with 62% in summer (April to September), with extremes observed near the end of these periods. Maximum detection occurred in February 2010 (100%), and minimum detection (46%) was measured in September 2010.

## Burden of Shellfish-Associated NoV Infection

Fish and shellfish represent 29% of NoV infections where food is a vehicle of infection in the UK (Table 4). The uncertainty regarding infection rates (as discussed above) is also important for shellfish. A comparison of 58 studies found that 8 NoV outbreaks (14%) were attributed to pre-harvest contamination of oysters, and a further 3 outbreaks (5%) to pre-harvest contamination of mussels and clams (Mathijs et al. 2012). In the UK, 16% of food-borne outbreaks were attributed to pre-harvest contamination of oysters from outbreak data between 1992 and 2000 (ACMSF 2014). In England, 289 outbreaks were reported to Public Health England (PHE) between 1992 and 2014, where seafood was recorded as a possible vehicle of infection (Table 5). Specifically, 120 of these outbreaks linked oysters as a vehicle of infection, affecting 1946 people. This is an average of 85 people (median 42) a year during this period and equates to ~ 16 people for each outbreak. In the UK, the potential number of community cases infected by consumption of contaminated shellfish was estimated at between 14,593 and 30,160 cases per year, with some of these cases leading to ongoing transmission (calculated from Table 3 and Table 4).

**Table 4:** Estimated fraction of norovirus transmitted via different food categories

Food category	Proportion of food-borne norovirus (%)			
	UK^a^	Netherlands^b^	Canada^c^	USA^d^
Fish and shellfish	29	34.7	35.7	35.6
Poultry	16	6.5	2.2	1.6
Composite and ‘other’ foods	16	10.9	7.9	0.2
Fruit and vegetables (produce)	12	15.2	31.5	39
Pork	11	6.5	2.3	1.5
Eggs	7	4.3	0.9	1.1
Grains and beans	7	10.8	4.3	6.1
Unspecified red meat and game	1	0.2	9.9	10.4
Beef and lamb	0.5	6.5	2.7	1.5
Dairy products	0.5	4.3	2.5	3

Adapted from: ACMSF (2014)

^a^Tam et al. (2014)

^b^Havelaar et al. (2008)

^c^Davidson et al. (2011)

^d^Hoffman et al. (2007)

**Table 5:** Number of outbreaks reported to public health England associated with the consumption of shellfish and crustacean between 1992 and 2014 (PHE 2015)

Year	Outbreaks			Number of individuals affected	
	Seafood	Shellfish/crustacea	Oysters	Number affected (crustacea/shellfish)	Number affected (oysters)
1992	17	14	10	324	183
1993	14	10	5	203	74
1994	20	11	7	125	104
1995	26	15	7	869	76
1996	21	8	5	254	81
1997	30	14	10	182	100
1998	11	6	3	156	42
1999	14	4	2	53	27
2000	11	7	5	164	32
2001	9	5	3	46	21
2002	2	1	1	7	7
2003	2	2	1	7	3
2004	7	5	3	108	37
2005	15	9	8	126	92
2006	12	11	9	186	44
2007	5	2	2	12	12
2008	6	5	4	26	24
2009	16	11	11	732	732
2010	15	13	11	132	101
2011	9	7	4	59	32
2012	6	4	1	30	13
2013	13	13	5	329	91
2014	8	6	3	166	18
Total	289	183	120	4296	1946

**Fig. 2:**
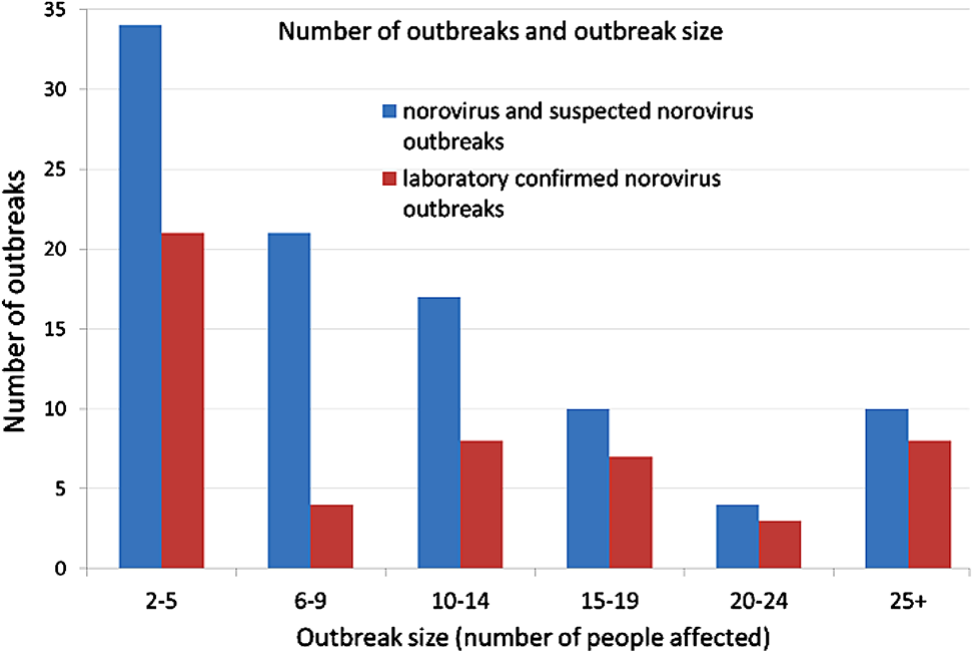
Outbreak size for confirmed and suspected norovirus outbreaks associated with oysters between 1992 and 2014 (PHE 2015)

**Fig. 3:**
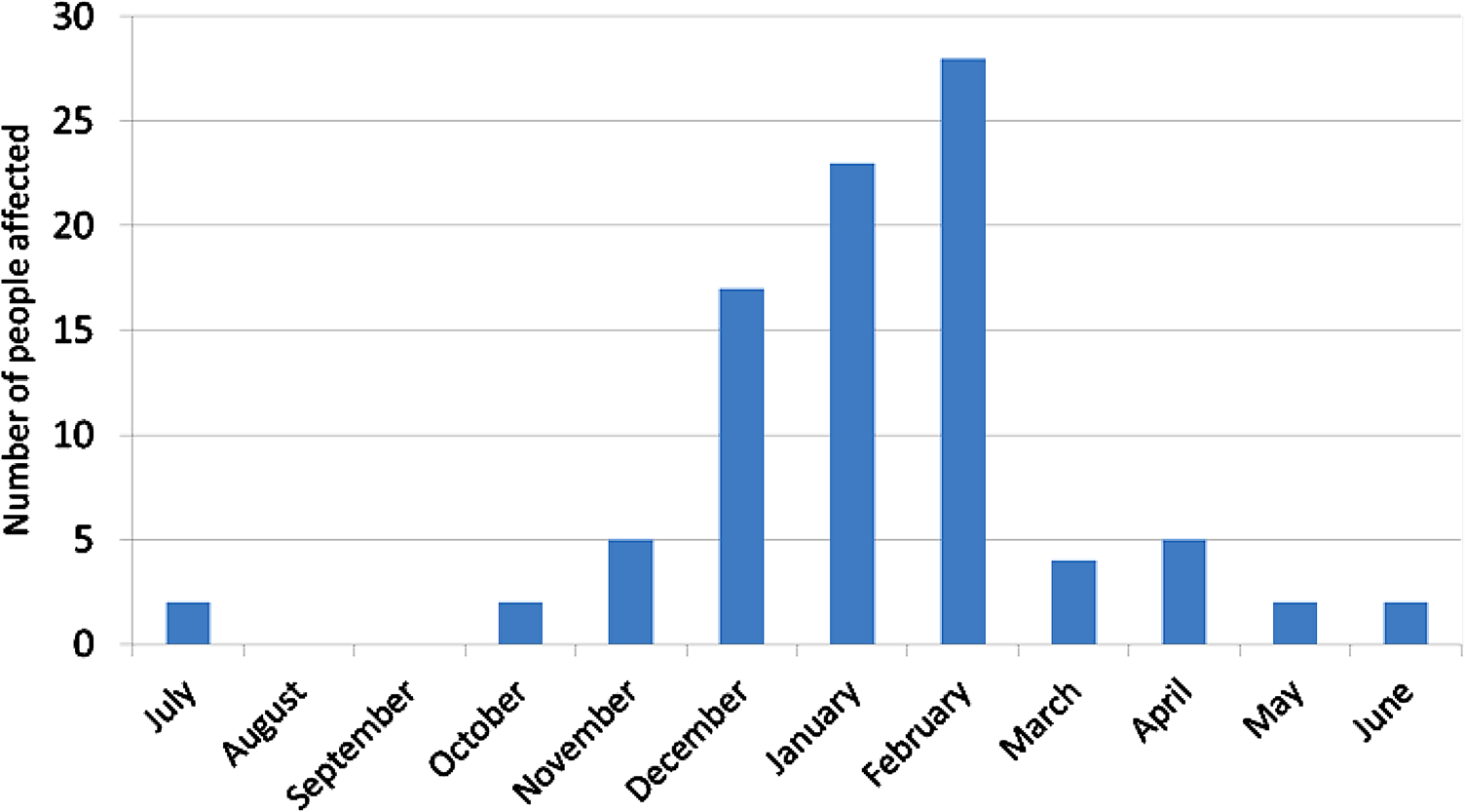
Number of people affected by gastrointestinal infection outbreaks reported where oysters are the implicated food product, by month of outbreak, between 1992 and 2014 (PHE 2015)

Most of the outbreaks reported to PHE did not have confirmation of NoV as the causative agent, due to a historical lack of diagnostic tools for NoV (Fig. 2). Outbreaks where people visit restaurants and are not known to one another and subsequently fall ill are rarely visible to the authorities, unless large numbers of people report illness (Lowther et al. 2010b). A recent study found that secondary infections occurred in 20% of individuals who did not consume shellfish from a restaurant outbreak (attributed to shellfish) highlighting the role of person-to-person transmission in shellfish cases (Alfano-Sobsey et al. 2012). The numbers of people affected by gastrointestinal oyster implicated outbreaks is generally < 5 people in non-winter months (March–November), and between 16 and 28 persons in winter months (December–February) (Fig. 3). However, attributing oysters as the vehicle of infection to an outbreak is challenging due to other possible vehicles of infection such as surfaces or person-to-person contact (Hall 2012; Repp and Keene 2012).

It is widely recognised that the burden of infectious disease is likely to increase in response to a range of factors including climate change, population growth, changes in diet and water supply, and the emergence of more virulent pathogens (Semenza and Menne 2009). It is predicted that flooding may increase with climate change and result in greater discharge of untreated human-derived wastewater into the coastal zone, although the impact on shellfish production requires further attention. A recent modelling study predicted that a doubling of NoV cases associated with food from 2.5% to 5% (of total cases) could increase the overall disease burden by up to 33%, due to the non-linear nature of ongoing transmission (Lane 2014). In addition, a reduction of fish and shellfish infections (33% of food mediated infections) would result in a 16% drop in total NoV infections suggesting efforts to reduce this vector would be useful. Therefore, based on these data preventing person-to-person spread would have the greatest impact on reducing NoV infections and reduce the overall burden of the disease. Consequently, the risk of contracting an IID after shellfish consumption was estimated at 646 cases per 1 million servings (0.00065%), the highest of all food groups. Due to their high viral load and raw consumption, oysters were specifically identified as posing a greater risk factor for NoV infection in adults (Phillips et al. 2010). Tam et al. (2012a, b, c) reported that in ~ 50% of all IID community cases, individuals took sickness leave due to their symptoms. Assuming an average loss of two days per symptomatic individual, this represents a total of between 14,593 and 30,160 days per year for shellfish-borne NoV infections (Table 6). This is likely to be an underestimate, due to underreporting and as food industry and hospital employees require longer before returning to work. Details of mortality due to NoV specifically from shellfish have not been categorically reported in the literature.

**Table 6:** Estimated community cases, GP consultations, and hospital admissions related to shellfish-borne norovirus transmission in the UK (2009)

Modelling approach	Community cases	GP consultations	Hospital admissions
	Cases (95% CI)	Cases (95% CI)	Cases (95% CI)
Monte Carlo	21,292 (14,593–30,160)	940 (576–1497)	136 (78–226)
Bayesian	21,489 (17,734–26,001)	950 (650–1371)	96 (72–128)

Calculated from: ACMSF (2014) and Tam et al. (2014)

Assumes a shellfish-borne rate equivalent to the rate of seafood-borne infection

Numerous outbreaks have been reported from fresh and preserved shellfish (Alfano-Sobsey et al. 2012; Webby et al. 2007). For example, Hewitt and Greening (2004) found that the standard pickling procedure did not reduce the viral gene copies (gc) for NoV or hepatitis A virus. However, a 1.7 log_10_ reduction in the hepatitis A virus infectivity in tissue culture infections was noted due to pickling. High hydrostatic pressure treatments have been suggested to reduce the infective load of NoV. Leon et al. (2011) found that 600 MPa completely inactivated spiked human NoV in oysters and did not cause infection in human volunteers. However, shellfish treated in this way are not sold live and have a lower economic value (Ye et al. 2014). Flannery et al. (2014) found that cooking time was critical for the reduction in infectious FRNA bacteriophage (as a surrogate for infectious NoV) in mussels. Freezing shellfish has been shown to be insufficient at reducing viral load and has been implicated in a number of outbreaks, e.g. frozen oysters have been implicated in 83 individual cases of gastrointestinal illness (Webby et al. 2007).

## Detection and Quantification of NoV

Despite significant efforts, NoV cannot be reliably cultured in vitro (Jones et al. 2015). Historically, NoV antigens have been detected using enzyme immunoassay (Farkas et al. 2006), although these techniques require strain-specific antibodies which cannot detect low concentrations of NoV present in shellfish (Schultz and Myrmel 2013). The development of RT-qPCR methods has allowed the reliable detection and quantification of NoV in food. However, shellfish represent a challenging matrix for reliable RT-qPCR quantification. Recently, a standard method for the detection and quantification of NoV in shellfish by RT-qPCR has been developed and utilised by the European Committee for Standards (Lees 2010) and was found to be reproducible in a European laboratory ring trial (CEFAS 2011a, b). Therefore, this method is suitable for detection and quantification of NoV for use in the application of viral hygiene standards (EFSA 2012). However, RT-qPCR has a number of drawbacks for quantification of NoV in shellfish which are discussed below.

### Limitations of Current Detection Approaches

Quantification of NoV RNA genome copies in a sample highlights the presence and concentration of NoV, providing evidence of the potential for disease. However, the detection of NoV genomes in a sample does not provide information on the infectivity of the viral particles from which they originate, and it is therefore difficult to assess the actual human health risk. Conventional RT-qPCR for NoV is limited by the reproducibility of virus or nucleic acid extraction, the presence of reverse transcription and PCR inhibitors in the matrix, and the high genetic variability of NoV (Le Guyader et al. 2006). A comparison of extraction methods showed that proteinase K digestion followed by NucliSENS miniMag extraction was the most efficient method for NoV recovery (Uhrbrand et al. 2010). However, an average recovery of 1% and 2% from mussels and oysters, respectively, was achieved. Therefore, the corrected (to account for extraction efficacy) NoV values would be of a greater titre than uncorrected values, with important considerations for risk assessment and proposed shellfish standards (Petterson et al. 2015).

### Approaches to Assess NoV Infectivity/Viability

NoV nucleic acid associated with inactivated viruses can still remain detectable by PCR after the virus is no longer infective. For cultivable examples (e.g. Hepatitis A virus), virus particle concentrations (genome copies) were 10- to 1000-fold higher than the plaque-forming unit (infective particles) concentrations of the same sample (Li et al. 2011). Consequently, discriminating infectious from non-infectious NoV represents a critical research gap where progress is necessary to apportion the human health risk of shellfish (Knight et al. 2012). Several alternative approaches for assessing NoV infectivity are currently being investigated and include those based on examining the integrity of the virus genome, those examining capsid and/or binding site integrity (Knight et al. 2012) and those based on viral culture on susceptible cell lines. The ability to culture NoV from environmental samples requires substantial research effort and although NoV infection in vitro human cell lines has been achieved, further optimisation is needed to enhance the efficiency of the method (Jones et al. 2015; Ettayebi et al. 2016). Specifically, it should be noted that while these newly developed cell line assays work well with pure viral cultures, inoculation with environmentally derived NoV poses problems from other contaminants present in the sample. A review on candidate methods to assess infectivity of viruses is provided by Knight et al. (2012).

## Regulating NoV in Shellfish Waters and Shellfish: Current Practice and Future Options

### Shellfish Quality Assessment

Bacterial contamination of harvested shellfish is regulated under EC Food Hygiene Regulations (e.g. Regulations 854/2004 and 1021/2008), under which standards of compliance based on FIBs determine if shellfish can be harvested. Shellfish hygiene regulations currently do not include virus contamination. However, viruses such as NoV and Hepatitis A (HAV) have been detected in shellfish (Polo et al. 2015), which were implicated in NoV and HAV outbreaks (Loury et al. 2015). To ensure protection of shellfish waters, the introduction of virus testing methods or viral standards has been proposed to improve viral hygiene with respect to shellfish production, and is discussed below (EURL 2014a).

### Legal Obligations Relating to NoV in Shellfish Waters

The obligations of UK water companies relating to potential impacts on shellfisheries are regulated based on legislation derived from EU directives. Wastewater effluents are regulated via a set of standards which limit pollutant concentration in the discharge (i.e. treatment of influent and an assessment that the receiving water body has the capacity to disperse the polluted effluent, without significant impact or deterioration) (DCWW 2015). Shellfish waters are regulated within the Water Framework Directive 2000/60/EC (WFD) (EC 2000). The WFD requires that there is no deterioration in water quality in coastal and brackish waters supporting shellfish (bivalves and gastropod molluscs) suitable for human consumption. In 2009, shellfish waters were uniformly designated as ‘Protected Areas’ under the river basin management plans, the Directive requires WWTWs with significant contributions to the pollution of shellfish waters to be treated using tertiary technologies regardless of the population size served by WWTWs draining into the production area (DEFRA 2012). The majority of intermittent CSO or SO discharges do not have a legal requirement for microbial standards prior to discharge. However, some monitored CSOs requiring a permit for discharge and tertiary treatment (e.g. UV) can be a requirement of consent.

### End-Product Shellfish Testing

Until 2013, standardised detection methods for viral pathogens were not available for routine analysis for shellfish. End-product testing for viruses by RT-qPCR and viral standards are being considered in addition to existing sanitary measures for shellfish harvesting areas (EFSA 2012). A challenge to the implementation of a viral standard is the extent to which NoV RNA detected by RT-qPCR is correlated with viral infectivity or risk of human illness. However, there is some evidence linking the risk of illness from consuming oysters containing NoV concentrations exceeding 2000 gc g^−1^, while outbreaks have been associated with values of 1000 gc g^−1^ (Lowther et al. 2010a; FSAI 2013). However, these studies were based on self-reported customer illness complaints and rarely from confirmed cases. Further work could assess the probability of shellfish being the vehicle of infection via combined testing of shellfish and stool samples for confirmation of NoV as the likely source of infection.

In volunteer studies, ingestion of an inoculum containing 10^3^ gc g^−1^ of aggregated NoV particles was linked to a 60 ± 20% chance of infection. Counter to expectation, dispersed NoV ingested at an estimated dose of 10^7^ gc g^−1^ was also linked to a 60 ± 20% chance of illness (based on modelled data) (Teunis et al. 2008). Atmar et al. (2014) measured the 50% human infectious dose for susceptible individuals as 1300 gc (95% confidence, 440–3760 gc). The high variability in these estimates and low sample sizes used do not at present provide enough confidence to support the defining of standards for risk assessment purposes. Furthermore, the risk associated with the consumption of oysters containing low levels of NoV (e.g. <1000 gc g^−1^) has also not been reliably quantified. Understanding the relationship between dose and response is urgently required to underpin the principle of a quantitative standard. To date, this has proved problematic for NoV due to uncertainty in the degree of viral inactivation, viral aggregation, and differences in host susceptibility (Teunis et al. 2008; Atmar 2010; Atmar et al. 2014). A presence/absence standard may avoid some shellfish-borne illness. However, the presence of NoV does not confirm infectivity, and the cost for regulation, implementation, and enforcement is not proportionate to the health risk. Furthermore, as a high percentage of UK oysters are positive for NoV (estimated at 76% in Lowther et al. 2012b), a presence/absence standard would therefore not permit sale of most UK oysters.

The proposed quantitative limit for NoV in shellfish products placed on the market is 200 gc g^1^ (EURL 2014a). This level is thought to be consistent with the methodological constraints, and is possible for producers to achieve (Doré et al. 2010). The limit of 200 gc g^−1^ is the sum of two NoV genogroups (GI and GII), which in practice results in an action threshold close to, or below, the limit of quantification of the RT-qPCR assay (<100 gc g^−1^) (EURL 2014a). Viral standards may only be applied to shellfish products destined to be consumed raw due to thermal inactivation of viruses (Richards et al. 2010; Flannery et al. 2014).

End-product testing cannot reliably ensure that unsafe food is not placed on the market. The cost of end-product testing is placed on the food business operators (FBOs) (e.g. shellfish producers). A recent estimate suggested that end-product testing could cost producers 10% of the total value of their catch (Hess 2010); however, this value could be higher in smaller species of low economic value (e.g. mussels). In addition, end-product testing does not consider the root cause of the problem, which is human faecal pollution in the shellfish production area. Shellfish producers are also responsible for risk assessment in relation to production site and practices which are designed to minimise the impact of sources of pollution (DEFRA 2015).

### Production Area Testing for NoV

Production area testing is implemented in Europe to designate harvesting zone quality, based on faecal indicator bacteria (FIB). Depuration is used to reduce bacterial contamination in bivalve molluscs harvested from class B shellfish production areas. However, evidence suggests that NoV (and other enteric viruses) are not sufficiently removed by standard depuration practices (Polo et al. 2014). In addition, viral outbreaks have also been associated with shellfish harvested from class A production areas (EURL 2014a). A proposed NoV limits of 1000 gc g^−1^ have been suggested for shellfish production areas as this level is likely to result in an NoV concentration below the proposed 200 gc g^−1^ end-product limit (EURL 2014a). The cost of shellfish production area testing would be borne by the local competent authority (EURL 2014a). The use of viral limits, either in end-products or in the production area, could also be linked to the application of a label advising the public to cook the product, which would be mandatory to suppliers (EFSA 2012; EURL 2014a).

### Post-harvest Treatment of NoV in Shellfish

Post-harvest treatments (e.g. depuration) are required for commercially harvested shellfish that do not to meet required hygiene standards (EC 2004). Commercial shellfish beds in the UK are subject to hygiene classification by the Food Standards Agency (FSA) based on *E. coli* standards from EC (2004). In the most recently available hygiene classification listings (FSA 2015; FSAS 2014), the majority (74.2%) of UK oyster beds were classified as class B or C, and a further 4.2% were seasonally classified as class B in Scotland. Consequently, most UK-grown oysters must undergo post-harvest hygiene treatment, irrespective of viral load. Methods to purify shellfish are effective at reducing bacterial loading, but their efficacy at reducing viral loading to non-infectious levels may be limited (Neish 2013). Recommended purification treatments include depuration, relaying, and heat treatment. Depuration is the purification of shellfish by allowing natural purging of contaminants in a controlled environment by circulating clean water (Aquatic Water Services Ltd. 2014). Depuration treatments of between 1 and 4 days, using standard commercial conditions (Neish 2013), are used to effectively reduce bacterial contamination of oysters; however, their ability to reduce viral load has been criticised (Le Guyader et al. 2006).

Methods to enhance depuration include adjustments to water temperature, salinity, dissolved oxygen content, turbidity, and phytoplankton content, or the application of chlorination, UV irradiation, treatment with ozone and activated oxygen, or iodophors (Schneider et al. 2009). Using an animal cell line assay, UV treatment has been shown to reduce NoV surrogates to non-infectious levels (Garcia et al. 2015; Neish 2013). Typically, however, water temperature was elevated in these experiments and therefore the relevance to human infectivity is thought to be limited (Cook et al. 2015). Other studies using viral surrogates suggest the potential for reducing NoV loading using a salinity of ≥25‰ (FSA 2003), chlorination (de Abreu Correa et al. 2012), UV irradiation for extended depuration periods (Marques Souza et al. 2013), or dosing with antipathogenic bacteria (Fajardo et al. 2014). However, the validation of these methods has mainly been undertaken with surrogate viruses rather than NoV, restricting their applicability (Cook et al. 2015). Recently, water depuration between 15 and 17 °C has been shown to be effective at reducing NoV loading in shellfish (Doré et al. 2010; FSA 2003; Neish 2013). In contrast, ozone and UV irradiation have proven ineffective for NoV depuration at normal temperatures (Neish 2013).

Relaying oysters into a class A shellfish area for a minimum of two months is used to reduce FIB loading in oysters. However, the effectiveness of this strategy for NoV is linked to ambient viral loading in the relay area, NoV persistence in bivalve tissue, and recirculation to other batches of oysters. The FIB levels used to classify production areas rarely correlate well with shellfish viral loading (Brake et al. 2014). However, movement to Class A beds has proven effective in eliminating NoV from shellfish (Doré et al. 2010; Keaveney et al. 2009; Lowther et al. 2012a). In practical terms, the low availability of Class A beds and the economic costs of relaying are likely to prevent the commercial adoption of this approach.

### Potential of Implementing Changes to the Classification System

Under class B shellfish hygiene criteria, up to 10% of samples can be contaminated with up to 46,000 *E. coli* 100 g^−1^ shellfish flesh (bacteria quantified by most probable number or colony forming units). Shellfish contaminated with this level of faecal contamination pose a significant risk of elevated viral contamination. While this level of *E. coli* contamination may be depurated successfully under standard conditions, the corresponding viral contamination is unlikely to be depurated under standard conditions (Polo et al. 2014). It has been suggested that reducing this 10% tolerance for high-risk species such as oysters would increase bivalve shellfish safety (EURL 2014a). However, this strategy would impact producers by reclassifying marginal areas from class B to class C. However, this is unlikely to reduce the NoV risk as NoV has been associated with class A shellfish areas (EURL 2014a).

### Minimum Closure Period During High-Risk Events

After a significant faecal pollution incident, or known NoV outbreak, the competent authority may close the shellfish production area. Reopening of the production area usually occurs when the shellfish comply with *E. coli* standards. However, NoV outbreaks have been reported from shellfish production areas which have been reopened in these circumstances (EURL 2014a). Therefore, a minimum closure period following faecal pollution incidents or outbreaks caused by enteric viruses may reduce the occurrence of further outbreaks (EURL 2014a). In the USA, a minimum closure approach is implemented using a 21-day minimum period (NSSP 2013). Further, the French Food Authority Ministry (DGAI) requires that following a NoV outbreak the production area linked to the outbreak is closed for up to 28 days, with consistent negative NoV results and compliant bacterial indicators resulting in early reopening (CEFAS 2013). Further evidence is required to ascertain if shellfish pose a human health risk during a potential closure period.

### Active Management Systems

Active management of shellfisheries is currently being assessed, whereby closure and reopening of shellfish beds is based on pre-determined environmental trigger points. Predictive modelling of FIB load and the dynamic closure of commercial shellfisheries during this period can be used to prevent harvesting of contaminated shellfish. Subsequently, the shellfish production areas would reopen after contamination has returned to background levels, significantly reducing the period of time through which higher levels of controls are applied. This approach is gaining traction as a means to reduce the impact of longer-term closures of production areas on the shellfish industry and avoids the permanent closure of exclusion zones around discharges. However, the technical and economic viability of such schemes require demonstration and appropriate triggers for closure are likely to vary significantly between catchments, requiring adaptive design, and validation for each shellfish production area. Active management systems have been considered as a supplementary approach to current shellfish waters classification based on FIB contamination levels, but direct applicability to management of NoV contamination is limited as different environmental triggers are likely to apply and due to the lack of an effective method for measurement of infective viral loading in shellfish that would be required to establish thresholds for closure.

### Shellfish Harvesting Exclusion Zones

A number of different types of exclusion zone could be considered, including geographical- proximity-based zoning, dilution-based zoning, dilution/time-based zoning, and shellfish sampling-based zoning (Aquatic Water Services 2014). Proximity-based zoning has been used to exclude harvesting in areas ranging from 50 to 1500 m from discharges or other inputs (Aquatic Water Services Limited 2014). However, the efficacy of geographical zoning for reducing NoV risk is strongly dependent on prevailing hydrodynamics (Winterbourn et al. 2016). Norovirus load in shellfish did not significantly decrease in ≤7 km from a point source discharge, despite *E. coli* shellfish concentrations meeting class A criteria (Campos et al. 2015). Dilution-based zoning is mandatory for all conditionally approved areas in the USA of 1:1000 dilution of sewage effluent to protect from viral contamination of harvesting zones (NSSP 2013). Under the NNSP model, reopening of shellfish beds could occur earlier than the 21-day closure following an exposure event via the use of bacteriophage reductions as a proxy (Aquatic Water Services Limited 2014).

A whole-system approach would require a comprehensive NoV survey to inform the zone scaling (Aquatic Water Services Limited 2014). However, in the absence of this information, a hybrid solution is possible, whereby an *E. coli* proxy is used for NoV risk (Petterson et al. 2015). This proxy could be linked to target NoV standards for harvest, and could be adjusted accordingly on the advent of new information.

### Implications for the Adoption of Viral Standards

The European Commission (EC) has proposed that a formal standard for NoV could improve the hygiene of shellfish destined for human consumption (EURL 2014a). As described above, the proposed limits for NoV are 200 genome copies per gram of digestive tissue (gc g^−1^ dt) for end-product, or harvest standards of shellfish collected from the seabed, for which the limit is proposed to be a maximum 1000 gc g^−1^ dt (CEFAS 2013). Standards relating to NoV contamination will have socioeconomic implications for bivalve aquaculture and this warrants further investigation (FSA 2003; Oliveira et al. 2011). The costs associated with viral inactivation are likely to vary between shellfish species, since NoV prevalence has been shown to vary between species even when grown at the same location (Polo et al. 2015; Suffredini et al. 2014), in addition to differential infectivity of NoV to humans (Le Guyader et al. 2010). Potential modes of compliance for the water sector could include exploring emergent technologies for viral removal, increasing UV dose/efficacy for UV tertiary treatments, installation of UV polishing on CSOs, and pipe relocation to increase the dilutant rate prior to contact with shellfish beds (DCWW 2015). To justify significant investments, a robust assessment of potential socioeconomic and environmental costs and benefits/disbenefits for each of the proposed categories of viral standards, for different regions at different times of the year, would be advisable (WHO 2006). Implementation of certain measures might also result in reduced compliance with other regulations; for example, installing or improving UV disinfection units could result in increased electricity consumption, in an environment where a reduction in energy consumption is encouraged (UK Parliament 2008). Novel disinfection technologies, e.g. pulsed or low-pressure UV systems or plasma treatments have shown to reduce NoV concentrations and infectivity of surrogates (Lee et al. 2016; Barret et al. 2016). Finally, logistical constraints limit the physical modification of some facilities; for example, UV disinfection unit installation at CSOs is only practicable at larger facilities where there is sufficient space for additional plant and infrastructure (DCWW 2015). Consideration of these and other potential barriers to implementation should be included within a robust cost–benefit analysis prior to implementation of proposed standards.

## Conclusions

In conclusion, NoV remains a truly global economic disease of which shellfish will remain a small but expensive infection vehicle to the human population for years to come. Norovirus contamination of shellfish and their production areas represents a significant challenge to the sustainable expansion of aquaculture in colder climates. The burden of NoV is likely to increase in response to climate change and population growth as increasing rainfall/runoff increase pathogen loading and survival in the environment. While the disease burden of shellfish-derived NoV is low, typically 646 cases per 1 million servings, it is the highest of individual food groups in the UK. Epidemiological efforts to apportion confirmed NoV infections to each food group is hampered by a high underreporting ratio and confounded by person-to-person transmission and contamination of food post-harvest. Shellfish will remain a significant vehicle of transmission of NoV to the human population, particularly as species such as oysters are eaten raw.

Recent efforts to standardise detection methods for NoV have improved quantification within food groups. However, both the differential extraction efficacy and co-extraction of inhibitors prevent reliable quantification between different food groups as vehicles for NoV infection. End-product testing of NoV in shellfish is problematic because pooled samples do not adequately protect the consumer from every animal in a batch. In addition, quantification of NoV gc g^−1^ of shellfish digestive tissue does not correlate to intact, infective viral particles. Legal and economic complications surround end-product testing as it does not guarantee the absence of NoV in unsampled shellfish, only that the sub-sample tested from a batch is free of NoV and could reduce the viability of shellfisheries.

Implementation of a shellfish standard for NoV (based on either end-product testing or harvesting areas) will not directly reduce the viral loading to the environment, but may reduce viral outbreaks in the community. As the majority of NoV infections are not attributed to shellfish, it is likely that community-level action is required to effectively isolate infected persons, particularly in hotspot areas such as hospitals and schools to reduce outbreaks through person-to-person contact. Antiviral disinfectants are not currently widely available for food preparation areas; however, conventional deep clean procedures (bleach and soap) are effective at reducing NoV. Product labelling, education in food preparation areas, and widespread advice from healthcare authorities offer routes to protect the public from NoV exposure. Improved understanding of the concentrations and infectivity of NoV in different vehicles of infection will enable identification of the risk of NoV going forward. Shellfish standards could be revisited once the relationship between infectivity and abundance in different vehicles of infection has been elucidated.

Control of NoV should reduce the risk prior to harvest, as the most significant sources of NoV in shellfish harvesting areas are point sources of human faecal pollution. Currently, water utilities in Europe are not regulated for viral pollution. However, the reliance on indicator bacteria does not adequately represent the risk, particularly from pathogenic viruses such as *NoV.*

Effective wastewater treatment, followed by viral inactivation technologies, is required to effectively reduce viral loading to the environment and therefore shellfish. However, it is not possible to recommend a single solution for inactivation of NoV, as on-site considerations and cost often override decisions regarding viral inactivation (Barret et al. 2016). The efficacy of current technologies for ameliorating viral pollution requires additional attention. Upgrade of existing wastewater treatment assets to technologies such as MBRs, anaerobic digestion, or tertiary technologies is often effective for reducing the viral load in the final effluents. The success of upgrades would be highly site specific and be contingent on financial restrictions, greenhouse gas, and effluent quality standards. Effluent pipe or shellfish bed relocation is the lower cost options which have proof of concept based on principals from dilution-based zoning. Effective reductions in CSO discharges, either through storm water transfer schemes, pipe relocation or increased wastewater storage and treatment prior to discharge, could reduce NoV loading to shellfish waters. However, costly infrastructure changes are likely to be driven through desire from water utilities to attain standards for bacterial bathing and shellfish water quality, due to monitoring, legislative, and enforcement administration which is currently in place, as opposed to NoV standards.
